# GSTP1 negatively regulates Stat3 activation in epidermal growth factor signaling

**DOI:** 10.3892/ol.2012.1098

**Published:** 2012-12-28

**Authors:** XIANJUAN KOU, NING CHEN, ZHIYONG FENG, LAN LUO, ZHIMIN YIN

**Affiliations:** 1 College of Health Science, Wuhan Institute of Physical Education, Wuhan 430079;; 2 Jiangsu Province Key Laboratory for Molecular and Medicine Biotechnology, College of Life Sciences, Nanjing Normal University, Nanjing, Jiangsu 210046;; 3 State Key Laboratory of Pharmaceutical Biotechnology, School of Life Sciences, Nanjing University, Nanjing, Jiangsu 210093, P.R. China

**Keywords:** glutathione S-transferase π, Stat3, phosphorylation, cell cycle

## Abstract

Glutathione S-transferases (GSTs) are the enzymes that defend cells against the damage mediated by oxidant and electrophilic carcinogens. GSTπ (GSTP1) is a member of the GST family and the hypermethylation GSTP1 CpG island DNA is detected in human hepatocellular carcinoma (HCC) tissues, which contributes to the negative expression of GSTP1 mRNA and protein. GSTP1 expression is considered to be an early event in HCC. Stat3, a member of the signal transduction and activator of transcription (Stat) family, is important for promoting the proliferation, survival and other biological processes of cells triggered by cytokines and growth factors. Activated Stat3 may participate in oncogenesis. Previous studies have demonstrated that overexpression of phosphorylated Stat3 is important in the proliferation of HCC cells, suggesting that disturbance of the Stat3 pathway may be an early event. We hypothesize that the suppression of GSTP1 expression in HCC cells increases Stat3 activation. In order to test this hypothesis, HepG2 cells were genetically modified to transiently express high levels of GSTP1. The transient expression of GSTP1 specifically downregulated epidermal growth factor (EGF)-mediated tyrosine phosphorylation of Stat3, and subsequently suppressed the transcriptional activity of Stat3. By contrast, GSTP1 RNAi was able to lead to an increase in the phosphorylation of Stat3. In addition, overexpression of GSTP1 was capable of reducing the survival of HepG2 cells and inducing cell cycle arrest. This inhibition was mediated by a direct interaction between GSTP1 and Stat3. Overall, our results suggest that GSTP1 is important in the regulation of the transcriptional activity of Stat3, and that it is also a regulator of the cell cycle via EGF signaling.

## Introduction

Glutathione S-transferases (GSTs), a superfamily of detoxifying enzymes, contain at least five subclasses, including α, *μ*, π, ω and θ. GSTs act catalytically through the nucleophilic attachment of the sulfur atom of glutathione (GSH) onto the electrophilic groups of substrate molecules (1,2). GSTs are important in protecting cells from cytotoxic and carcinogenic agents, removing oxidative stress products, and modulating cell proliferation and signaling pathways (2,3). As an isozyme of GST, GSTP1 is a major regulator of cell signaling in response to stress, hypoxia, growth factors and other stimuli. Previous studies have demonstrated that GSTP1 inhibits lipopolysaccharide-induced MAPK, and that NF-κB activation decreases LPS-induced iNOS production by regulating MAPK activation (4). In addition, GSTP1 expression is highly correlated with carcinogenesis; GSTP1 is overexpressed in a variety of human cancers, including lung, colon, ovary, bladder and kidney cancer (5–8). By contrast, the reduced expression and activity of GSTP1 are observed due to the hypermethylation of its promoter in hepatocellular carcinoma (HCC) and prostate cancer (9–10), although GSTP1 may also be detected in the corresponding non-tumorous tissues. However, GSTP1 null mice reveal an increased risk of carcinogen-induced skin tumorigenesis (11). Notably, the overexpression of GSTP1 has been reported to protect prostate cells from cytotoxicity and DNA damage due the heterocyclic amine carcinogen PhIP (12), which suggests that silencing of the GSTP1 gene by CpG island DNA methylation may be important in the development of HCC.

The signal transducer and activator of transcription (Stat) family of cytoplasmic proteins is important for promoting the proliferation, survival, and other biological processes triggered by cytokines and growth factors, including epidermal growth factor (EGF) (13–15). EGF induces the activation of Stat1, Stat3 and Stat5 in cancer cells. Stat3 has been demonstrated to play a critical role in EGF signaling in both normal and tumor cells (16). Normal Stat activation is a highly regulated process. However, atypical activation of Stat3 is usually detected in various human tumors including HCC, and may modulate the oncogenic transformation and progression (17). Furthermore, Stat3 has been implicated as a promising target for HCC therapy, as the inhibition of Stat3 has been shown to induce growth arrest and apoptosis of human HCC cells (18). Since GSTP1 exerts important anti-inflammatory, antioxidant and detoxification functions in the body, and its promoter is hypermethylated in HCC, the restoration of GSTP1 expression may be a promising method for preventing tumors. In the present study, the possible regulatory mechanisms of GSTP1 on Stat activation have been explored in HepG2 cells. The results indicate that the overexpression of GSTP1 specifically downregulates Stat3 activation, and inhibits cell growth via a direct interaction between GSTP1 and Stat3.

## Materials and methods

### Antibodies and reagents

The p-Stat3 (Y705), Stat3 and cyclin D1 antibodies were purchased from Cell Signaling Technology, Inc. (Beverly, MA, USA). A mouse monoclonal antibody against xpress-tag was purchased from Invitrogen Life Technologies (Carlsbad, CA, USA). Flag-tag was purchased from Sigma (St. Louis, MO, USA). GAPDH and protein G were purchased from Roche Applied Science (Indianapolis, IN, USA). Mouse or rabbit A/G and IgG antibodies were purchased from Santa Cruz Biotechnology, Inc. (Santa Cruz, CA, USA). Secondary antibodies coupled to IRDye 800 fluorophore for the Odyssey Infrared Imaging System were purchased from Rockland Immunochemicals, Inc. (Gilbertsville, PA, USA).

### Plasmid construction

Flag-Stat3 (wt) was provided by Dr Zhijie Chang of Tsinghua University (Beijing, China). GSTP1-RNAi was constructed into pRNA-u6. All expression plasmids were confirmed by sequencing and purified by the Endofree Plasmid Preparation kit (Qiagen, Hilden, Germany).

### Cell culture and transfection

HEK293, HepG2 and WRL-68 cell lines were purchased from the Institute of Biochemistry and Cell Biology, the Chinese Academy of Sciences (Shanghai, China), and then cultured in Dulbecco’s Modified Eagle’s Medium (DMEM; Invitrogen Life Technologies) supplemented with 10% fetal bovine serum (FBS; HyClone Laboratories; Logan, UT, USA), 100 U/ml penicillin and 100 *μ*g/ml streptomycin in 5% CO_2_ at 37°C. Transient transfection was performed using the Lipofectamine 2000 reagent (Invitrogen Life Technologies) according to the manufacturer’s instructions. In all cases, the total amount of DNA was normalized by the empty control plasmids.

### Immunoprecipitation and immunoblotting analysis

HEK293 cells were washed twice with ice-cold phosphate-buffered saline (PBS; pH 7.4) and lysed in lysis buffer containing 20 mM Tris (pH 7.5), 135 mM NaCl, 2 mM EDTA, 2 mM dithiothreitol (DTT), 25 mM β-glycerophosphate, 2 mM sodium pyrophosphate, 10% glycerol, 1% Triton X-100, 1 mM sodium orthovanadate, 10 mM NaF and 1 mM phenylmethylsulfonyl fluoride (PMSF), supplemented with complete protease inhibitor cocktail (Roche Applied Science). Following incubation on ice for 30 min, the cell lysates were centrifuged at 15,000 × g at 4°C for 15 min. Proteins (500 *μ*g) were immunoprecipitated with the designated antibodies, respectively. The precleared Protein A/G PLUS-Agarose beads (Santa Cruz Biotechnology, Inc.) were incubated with immunocomplexes for 2 h and washed four times with the lysis buffer. The immunoprecipitates were subjected to sodium dodecyl sulfate-polyacrylamide gel electrophoresis (SDS-PAGE), then transferred onto a nitrocellulose membrane (Hybond-C; Amersham Biosciences Corp.; Piscataway, NJ, USA). The immunoblotting analyses were performed. The results were visualized using IRDye 800 fluorophore-conjugated antibody in the Li-COR Odyssey Infrared Imaging System according to the manufacturer’s instructions (LI-COR Biosciences; Lincoln, NE, USA).

### Cell cycle assay

Cells were collected by trypsinization, pelleted at 800 × g for 10 min and fixed in 70% ethanol. The DNA content was evaluated by flow cytometry with propidium iodide (PI) staining. Flow cytometric analysis was performed using FACScan (Becton-Dickinson; Mountain View, CA, USA) with Cell Quest software.

### Cell viability

The transfected HepG2 cells were seeded in 96-well plates and the cell viability was evaluated by a 3-(4,5-dimethylthiazol-2-yl)-2,5-diphenyltetrazolium bromide (MTT) assay. For each experiment, six wells were used and the experiments were repeated three times.

### Statistical analysis

All experimental data was obtained from cultured cells were expressed as mean ± SD. Western blotting analysis experiments were repeated 3 times with similar trends. A one-way repeated measure analysis ofvariance and a Student’s t-test were used to determine the significance of the difference between two groups.

## Results

### Overexpression of GSTP1 inhibits EGF-induced Stat3 activation

**Figure 1:**
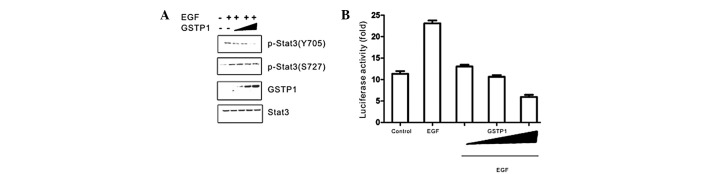
Overexpression of glutathione S-transferase π (GSTP1) suppresses epidermal growth factor (EGF)-induced Stat3 activity. (A) HepG2 cells were transfected with the xpress-tagged GSTP1 expression plasmid (0.5, 1 and 2 *μ*g). The cells were either left untreated or treated with EGF (100 ng/ml) for 15 min. Total cell lysates were prepared and subjected to western blot analysis using antibody against phospho-Tyr-705-Stat3 (p-Stat3 Y705) or phospho-Ser-727-Stat3 (p-Stat3S727). (B) GSTP1 suppresses EGF-induced Stat3 promoter activity. The HepG2 cells in 12-well plates were transiently transfected with different quantities of the xpress-tagged GSTP1 expression plasmid together with the pCMV-β-gal control vector and Stat3 reporter plasmid. Twenty-four hours following transfection, the cells were stimulated for 15 min. Cell lysates were prepared to measure luciferase activity using the Luciferase Assay System (Promega Corporation; Madison, WI, USA) and analyzed by the Luminometer TD-20/20 (Turner Biosystems, Inc.; Sunnyvale, CA, USA). Luciferase activity was normalized to β-gal activity.

In order to explore the effect of GSTP1 on endogenous Stat3 activation in HepG2 cells, the cells were transfected with xpress-tagged GSTP1 followed by EGF stimulation. The phosphorylation of Stat3 was examined by western blot analysis. Overexpression of GSTP1 inhibited the EGF-stimulated tyrosine phosphorylation of Stat3 in a dose-dependent manner (Fig. 1A). However, serine phosphorylation of Stat3 and a change in the expression of Stat3 were not observed. In order to explore whether GSTP1 is able to modulate Stat3 transcriptional activity in the presence of EGF, HepG2 cells were co-transfected with Stat3-dependent luciferase reporter gene and an xpress-GSTP1 plasmid. As is demonstrated in Fig. 1B, the cells were stimulated by EGF for 15 min and a 3-fold enhancement in fluorescence intensity was observed when compared with the control cells. However, the increase of fluorescence intensity was blocked in the presence of exogenous GSTP1. These results indicate that the suppression of Stat3 transcription may result from the inhibition of its tyro-sine phosphorylation.

### GSTP1 knockdown increases tyrosine phosphorylation of Stat3 stimulated by EGF

**Figure 2:**
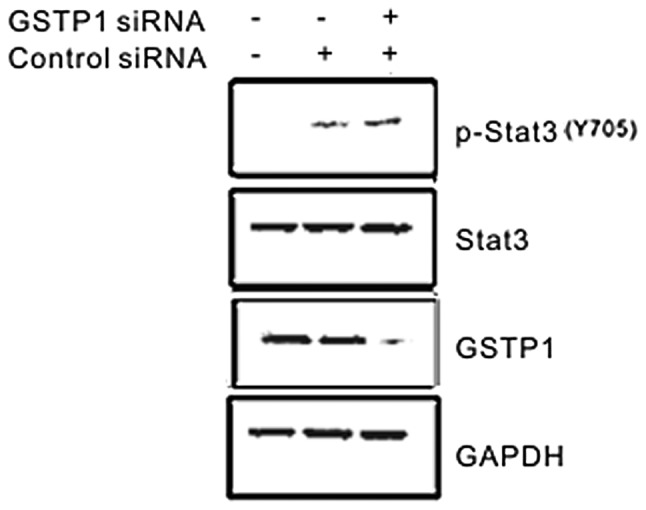
Knockdown of endogenous glutathione S-transferase π (GSTP1) expression enhances tyrosine phosphorylation of Stat3 stimulated by epidermal growth factor (EGF). WRL-68 cells were transfected with 40 *μ*l of 20 *μ*M siRNA of GSTP1 or the control siRNA duplex with Lipofectamine 2000 reagent for 12 h. Cells were transferred to a normal culture medium for 14 h and treated with EGF for 30 min before harvesting.

In order to further confirm whether GSTP1 downregulated the phosphorylation of Stat3, the GSTP1 siRNA was transfected into WRL-68 cells that have higher endogenous levels of Stat3. The effect of GSTP1 siRNA on EGF-mediated tyrosine phosphorylation of Stat3 was examined. As expected, the expression of GSTP1 was effectively blocked by GSTP1 siRNA, and GSTP1 siRNA further enhanced the EGF-stimulated tyrosine phosphorylation of Stat3 (Fig. 2). By contrast, GSTP1 siRNA had no effect on the expression level of Stat3. These results indicated that endogenous GSTP1 negatively regulated EGF-induced Stat3 activation.

### Effects of forced expression of GSTP1 on the cell proliferation and cell cycle phase distribution in HepG2 cells

**Figure 3:**
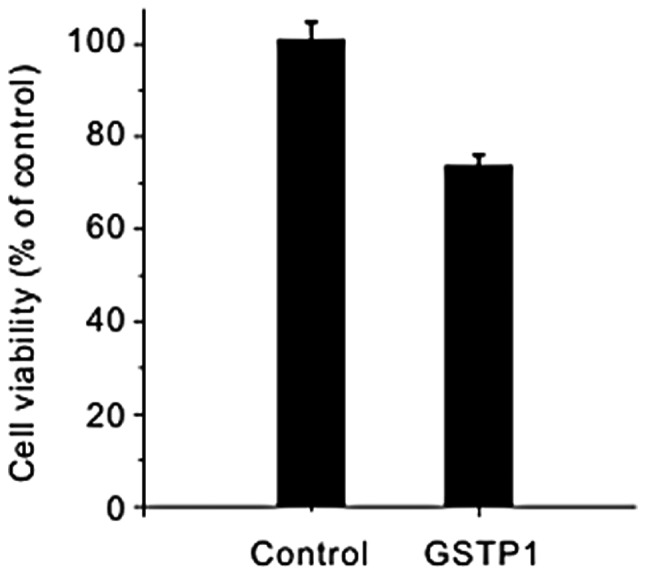
Glutathione S-transferase π (GSTP1) overexpression results in a reduction of proliferation and an increase in the number of cells in the G0/G1 phase. The HepG2 cells were transfected with empty vector and wild-type GSTP1 prior to treatment with epidermal growth factor (EGF). The extent of cell viability was assessed by the 3-(4,5-dimethylthiazol-2-yl)-2,5-diphenyltetrazolium bromide (MTT) assay. In each experiment, averages of eight replicates were normalized to the average of the vector control. Data are presented as mean ± standard error of the mean from ≥3 independent experiments.^*^P<0.05 compared with the vector control.

**Figure 4:**
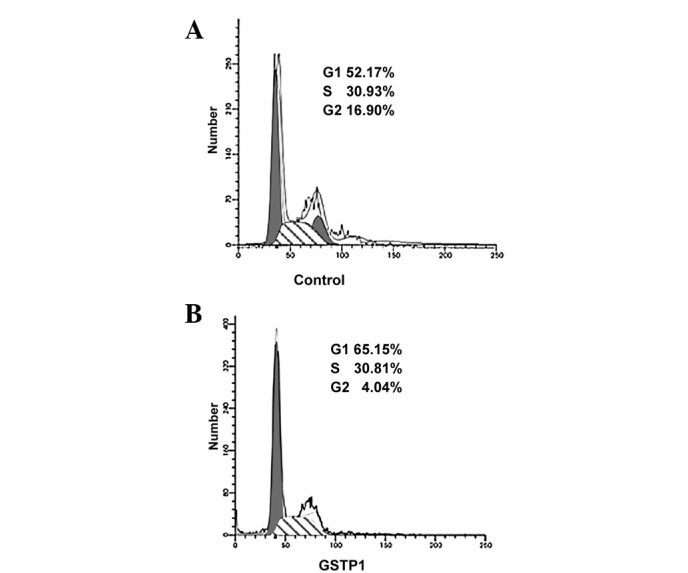
Glutathione S-transferase π (GSTP1) overexpression results in cell cycle arrest. The HepG2 cells were transfected with an empty vector and wild-type GSTP1 (1 *μ*g), collected 24 h after transfection, stained with propidium iodide (PI) and then analyzed by flow cytometry. The percentage of cells in each phase of the cell cycle is shown.

Since GSTP1 inhibits Stat3-dependent luciferase activity and previous studies have demonstrated that GSTP1 inhibits cell proliferation (19), the effect of GSTP1 on cell viability was also examined. The HepG2 cells were transfected with 2 *μ*g GSTP1 for 36 h and the GSTP1-transfected cells exhibited a reduced proliferation compared with the control HepG2 cells (Fig. 3), which suggested that GSTP1 possessed the function of suppressing cell proliferation. Additionally, Stat3 is important in cell cycle progression, whereas the inhibition of constitutively-active Stat3 induces cell cycle arrest (20,21). Flow cytometric analyses (Fig. 4) revealed that overexpression of GSTP1 induces cell cycle arrest and cell accumulation at the G0–G1 phase by 36 h when compared with untransfected cells. These results suggest that the GSTP1-induced inhibition of Stat3 signaling may result in the inhibition of cell growth and blockage of the cell cycle.

### GSTP1 expression inhibits Stat3 but not its upstream regulators

**Figure 5:**
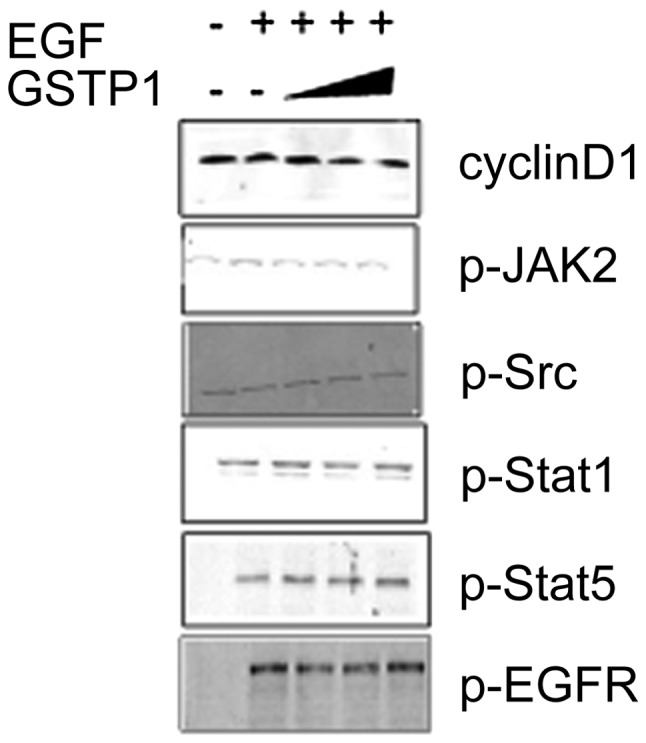
Effect of glutathione S-transferase π (GSTP1) overexpression on upstream kinases and cyclin D1. The cell lysates from HepG2 cells treated with GSTP1 for 48 h were resolved on 10% sodium dodecyl sulfate-polyacrylamide gel electrophoresis (SDS-PAGE), then immunoblotted with antibodies as indicated in Materials and methods. EGF, epidermal growth factor; p, phosphorylated: EGFR, EGF receptor.

It is well known that the phosphorylation of Stats depends on the activation of JAKs and/or Src family kinases, which stimulated us to explore the possible tyrosine kinases involved in the activation of Stats through GSTP1 inhibition. In order to gain insights into the inhibitory mechanism of GSTP1 on the Stat signaling cascade, the effect of GSTP1 on JAK and Src kinase activity was also examined. As demonstrated in Fig. 5, GSTP1 suppressed Stat3-mediated downstream factor cyclin D1, and did not affect the upstream regulators, such as p-JAK2, p-Src and p-EGFR, of the phosphorylation of Stat3. In addition, the phosphorylation of Stat5 was not affected by GSTP1, which revealed the specificity of GSTP1 for the phosphorylation of Stat3. Therefore, GSTP1 may inhibit the phosphorylation of Stat3 by direct inhibition at its protein level.

### Inhibition of Stat3 activity involves a direct interaction between GSTP1 and Stat3

**Figure 6:**
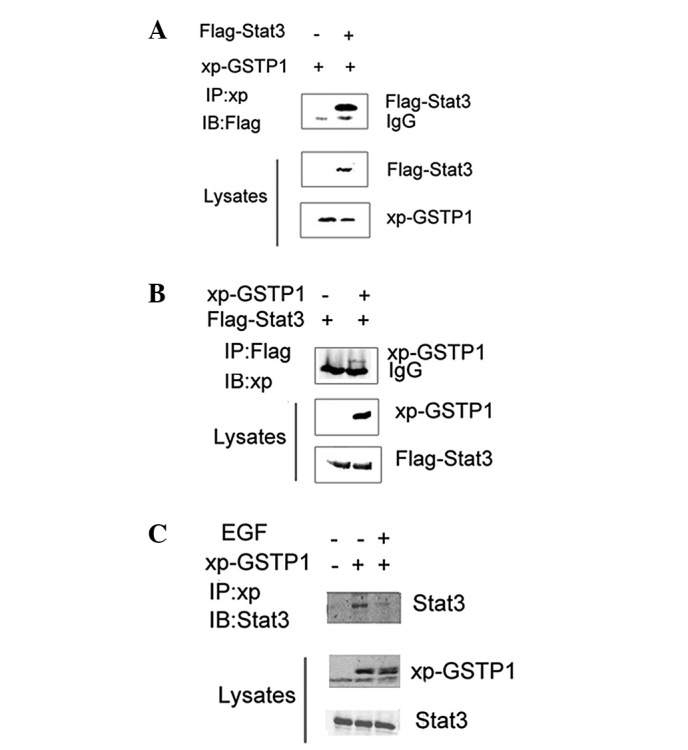
Glutathione S-transferase π (GSTP1) physically associates with Stat3. HEK293 cells were co-transfected with Flag-Stat3 (A) and xpress (xp)-GSTP1 (B) or empty expression vectors as indicated. Thirty-six hours following transfection, cell lysates were incubated with protein A/G PLUS-agarose beads conjugated with anti-xp antibody (A) and with protein A/G-Agarose beads conjugated with anti-FLAG antibody (B). The immuno-precipitated proteins were immunoblotted with anti-FLAG (A) or anti-xp (B) antibody. (C) xp-GSTP1 (1 *μ*g)-transfected HepG2 cell lysates were subjected to immunoprecipitation with anti-xp, and then the precipitates were analyzed by immunoblotting with anti-xp antibody. The cell lysates were immunoblotted with anti-Stat3 antibody or anti-xp antibody. IB, immunoblotting; IP, immunoprecipitation; IgG, immunoglobulin G. Similar results were observed in three independent experiments. EGF, epidermal growth factor.

In order to understand the mechanism of suppression of Stat3 activation, HEK293 cells transiently co-transfected with Flag-Stat3 and Xpress-GSTP1 were subjected to immunoprecipitation with anti-Flag or -Xpress antibody to explore the association between GSTP1 and Stat3. The immunoprecipitates were separated by SDS-PAGE, and transferred to a nitrocellulose membrane. The results indicated that GSTP1 co-immunoprecipitated with Stat3 in HEK293 cells (Figs. 6A and B). Similarly, a specific association between GSTP1 and endogenous Stat3 was observed in Xpress-GSTP1-transfected HepG2 cells. The co-immunoprecipitation assay demonstrated that Xpress-GSTP1 was able to physically interact with endogenous Stat3 (Fig. 6C). These results suggest that the negative regulatory effect of GSTP1 on Stat3 is mediated by a physical interaction between the two proteins.

## Discussion

GSTs are a superfamily of detoxifying enzymes that catalyze the conjugation of reduced GSH via a variety of electrophiles. In addition to their catalytic functions, GSTs also serve as nonenzymatic binding proteins, interacting with various lipophilic compounds including steroid and thyroid hormones (22–25). Furthermore, GSTP1 also regulates important normal cellular functions through its interaction with a number of critical cellular proteins, such as transglutaminase 2 (TGM2), apoptosis signal-regulating kinase 1 (ASK1) and Fanconi anemia group C protein (FANCC) (25). These findings suggest that the diverse functions of GSTP1 may be determined by the interactions with its key partner proteins. The currently identified mechanisms of Stat3 inhibition include dephosphorylation, inactivation of JAK by suppressor of cytokine signaling (SOCS1) protein (26) and abrogation of DNA binding by the protein inhibitor of activated Stat (PIAS) (27). However, in the present study, the physical interaction between GSTP1 and Stat3 resulted in the suppression of Stat3 activity (Fig. 6). Notably, upon treatment of HepG2 cells with EGF, a disassociation of the GSTP1/Stat3 complex was achieved, suggesting that Stat3 forms a complex with GSTP1 and that EGF may release Stat3 from the GSTP1 binding complex. The mechanism whereby GSTP1 specifically interacts with Stat3 may present a novel means of therapeutic intervention in Stat3-driven tumors.

HCC is the most common type of primary liver cancer, particularly in developing countries. More than half of cancer patients are identified as having HCC in China (28). Previous studies have reported that Stat3 is a promising target for HCC therapy (18). GSTP1 is downregulated or absent in HCC due to the action of DNA methyltransferase. However, the clinical applications of nucleoside analogs used as DNA methyltransferase inhibitors are limited somewhat by myelosuppression and other potential side effects. In the present study, the restoration of the GSTP1 protein has been demonstrated to exert an anticancer effect by inhibiting the Stat3 signaling pathway in HCC cells. We have demonstrated that the overexpression of GSTP1 in HepG2 cells suppresses the tyrosine phosphorylation and transcription activity of EGF-inducible Stat3, as well as the gene expression of Stat3-regulated cyclin D1, thus resulting in the inhibition of proliferation and increased accumulation of cells in the G1/G0 phase (Fig. 4). JAK2 or Src may be common upstream effectors for the activation of Stat3. Our results demonstrated that GSTP1 only inhibited Stat3, but no JAK2-dependent mechanism was observed in the transfected cells (Fig. 5).

In summary, a novel function of GSTP1 in inhibiting EGF-induced Stat3 activation has been demonstrated. The GSTP1-Stat3 complex reduces proliferation and arrests the cell cycle by terminating Stat3 activity.
